# Real‐world comprehensive diagnosis and “Surgery + X” treatment strategy of early‐stage synchronous multiple primary lung cancer

**DOI:** 10.1002/cam4.5972

**Published:** 2023-04-20

**Authors:** Danting Zhou, Tianyu Yao, Xiaojie Huang, Fang Wu, Yi Jiang, Muyun Peng, Banglun Qian, Wenliang Liu, Fenglei Yu, Chen Chen

**Affiliations:** ^1^ Department of Thoracic Surgery The Second Xiangya Hospital of Central South University Changsha Hunan P.R. China; ^2^ Hunan Key Laboratory of Early Diagnosis and Precise Treatment of Lung Cancer The Second Xiangya Hospital of Central South University Changsha P.R. China; ^3^ Department of Oncology The Second Xiangya Hospital of Central South University Changsha Hunan P.R. China; ^4^ Department of Pathology The Second Xiangya Hospital of Central South University Changsha Hunan P.R. China

**Keywords:** ablation, early‐stage, EGFR‐TKIs, gene mutation, SBRT, surgery, synchronous multiple primary lung cancers

## Abstract

**Background:**

Diagnosing and treating synchronous multiple primary lung cancers (sMPLC) are complex and challenging. This study aimed to report real‐world data on the comprehensive diagnosis and treatment of patients with early‐stage sMPLC.

**Materials and Methods:**

A single‐center cohort study was carried out and a large number of patients with early‐stage sMPLC were included. A single‐ or two‐stage surgery was performed to remove the primary and co‐existing lesions. The “X” strategies, including ablation, SBRT, and EGFR‐TKIs treatment, were applied to treat the high‐risk residual lesions. Wide panel‐genomic sequencing was performed to assess the genetic heterogeneity of the co‐existing lesions.

**Results:**

A total of 465 early‐stage sMPLC patients with 1198 resected lesions were included. Despite most patients being histologically different or harboring different genetic alternations, about 7.5% of the patients had the same histological type and driver gene mutation changes, comprehensive re‐evaluation is thus needed. The “Surgery + X” strategy showed remarkable efficacy and safety in treating multiple lesions. Follow‐up data revealed that the T2 stage (*p* = 0.014) and the solid presence of a primary lesion (*p* < 0.001) were significantly related to tumor recurrence. And a T2‐stage primary tumor had a significantly higher rate of developing new lesions after the initial surgery (*p* < 0.001).

**Conclusions:**

In real‐world practice, histopathological and radiological evaluation combined with genetic analyses could be a robust diagnostic approach for sMPLC. The “Surgery + X” treatment strategy showed remarkable efficacy, superiority, and safety in the clinical treatment of early‐stage sMPLC.

## 1 INTRODUCTION

With the advent of high‐resolution computed tomography (CT) and lung cancer screening program, the number of patients diagnosed with synchronous multiple primary lung cancers (sMPLC) is increasing in clinical practice. A major challenge in managing these cases is determining whether co‐existing nodules should be diagnosed and treated as separate primary cancers or intrapulmonary metastases, as the treatment strategies and outcomes differ significantly between the two forms of the disease.^1^, ^2^ Histological differences among co‐existing lesions can serve as reliable indicators for the diagnosis of sMPLC. However, it would be rather challenging if the synchronous tumors were histologically the same or similar.^3^, ^4^, ^5^ Genetic alteration evaluation using next‐generation sequencing technology may be an optional diagnostic strategy for identifying sMPLC. Previous studies have shown that patients with sMPLC have higher mutational frequencies in genes such as EGFR, RBM10, KRAS, and ERBB2.^6^ The high heterogeneity among the multiple lesions was considered the most significant genetic characteristic of sMPLC.^7^

Surgical resection remains the most employed strategy for treating early‐stage sMPLC.^8^, ^9^ It has been reported that anatomical resection of the primary lesion, followed by limited resection of the co‐existing or residual lesions, might be a safer and more beneficial alternative for co‐existing lesions.^8^ The sublobectomy, mainly including wedge resection and segmentectomy, has been widely accepted as an alternative choice since it allows major conservation of pulmonary function, especially for early‐stage lung cancer presenting as ground‐glass opacities (GGO).^10^, ^11^ However, the therapeutic complexity of sMPLC should be fully recognized. There is a lack of optional and effective therapeutic strategies for high‐risk residual lesions after initial surgery, especially in patients who are intolerant to surgical resection. Recently, some novel approaches, such as stereotactic body radiation therapy (SBRT), ablation, EGFR‐TKI treatment, and immunotherapy, were eventually applied to the treatment of sMPLC. With very preliminary data, these explorations provided new insights into the comprehensive clinical treatment of sMPLC.

The present study summarized our center's real‐world data on the clinical diagnosis and treatment of patients with sMPLC. Based on multidisciplinary diagnostic analyses of sMPLC, a comprehensive treatment strategy entitled “Surgery + X” was provided to patients with sMPLC. The clinicopathological, diagnostic, therapeutic, genomic status. and prognostic factors were analyzed.

## 2 MATERIALS AND METHODS

### 2.1 Study population

Between April 2015 and December 2020, patients who underwent curative‐intent surgical resection for lung cancer were retrospectively reviewed, and a total of 582 patients were diagnosed with multiple primary lung cancer (MPLC) according to the Martini‐Melamed criteria.^4^, ^12^ Patients who had a malignancy history or received preoperative induction therapy were excluded. Demographic variables were collected from all patients, including age, gender, smoking history, TNM stages, histological types, and CT features. To better determine the radiological characteristics of the nodules, all the patients underwent a high‐resolution CT scan before the surgery. The GGO, mixed GGO (mGGO), and the solid lesion was defined according to previous studies.^13^, ^14^, ^15^ The size of the lesions was determined preoperatively based on a thin‐section CT scan with 1‐mm collimation. This study was approved by the Institutional Review Board of the Second Xiangya Hospital.

### 2.2 “Surgical + X” strategies for the treatment of sMPLC lesions

Surgical procedures for each patient with sMPLC were determined based on age, cardiopulmonary function, tumor size, CT features, lesions distribution, and comorbidities severity.^15^, ^16^, ^17^ In patients with ipsilateral lesions with tolerable cardiopulmonary function, a single‐stage surgery was performed to remove the primary lesion and the co‐existing lesions with a high risk of malignancy.^15^ Anatomical resection was performed to remove primary lesions, and wedge resection was applied to remove the co‐existing lesions located at the edge of the lung with a diameter of <2 cm.

In the cases with bilateral lesions, a two‐stage procedure was recommended, first on the side of the lesion with an advanced stage based on preoperative assessment. The time of the second operation was suggested based on the patient's condition (mainly including age, cardiopulmonary function, and tumor morphology). Systemic and sampling lymph node dissection was performed in cases with a lobectomy and sublobar segmental and wedge resections, respectively. The largest T and highest N stages of all lesions in each case were treated as the final comprehensive T and N stages. The highest stage of all lesions determined the comprehensive TNM stage according to the eighth TNM classification guidelines.^18^, ^19^ A high‐resolution CT scan routinely followed all patients every 6 months after surgery.

Despite curative intent resection, there were still some high‐risk residual lesions in some of the sMPLC patients, which were not fit for additional resection due to impaired pulmonary function, improper location, or other medical conditions. Our multidisciplinary team assesses the properties of these residual lesions based on previous study strategies, considering changes in radiologic and biological behavior. GGO or mGGO residual nodules larger than 0.8 cm in diameter, or those that gradually increase in size or density during follow‐up, were considered high‐risk or progressed residual lesions that need to be treated.

The “X” strategies, including ablation, SBRT, and EGFR‐TKIs treatment, were applied to treat the high‐risk residual lesions in the following situation: (a) a co‐existing lesion that had a high risk of malignancy but was improper for additional surgical resection due to impaired pulmonary function or other medical conditions after initial surgery; (b) a progressed residual lesion; (c) a newly emerged lesion, existed persistently and evaluated by the multidisciplinary team to be a high risk of malignancy. The selection of the “X” strategy should take into account the location, anatomical variations, CT features, and patients' willingness. For GGO lesions located in the outer 1/3–1/2 of the lung, CT‐guided percutaneous puncture microwave ablation is usually recommended. For lesions located near the bronchi and closer to the center of the lung, electromagnetic navigation bronchoscopy‐guided microwave ablation (ENB‐MWA) is a common choice. For lesions with a high risk of complications from ablation, SBRT is an alternative option. For patients with a primary EGFRm lesion that reached stage IB and with high‐risk factors for tumor recurrence, adjuvant EGFR‐TKI treatment was recommended.^20^, ^21^

### 2.3 Tissue samples and gene mutation analysis

Fresh frozen tissues were collected from each resected nodule. Genomic DNA was extracted using the QIAamp DNA Tissue Kit (Qiagen). The samples were subjected to wide panel‐genomic sequencing (pan‐cancer 1021‐gene panel, Geneplus Technology Inc.) at the coverage depth of 1800×. Germline DNA from peripheral blood mononuclear cells of the same patients was used as a reference. The detection and data analysis were performed as described previously.^22^, ^23^, ^24^

### 2.4 Statistical analysis

Continuous variables were presented as mean ± standard deviation, while categorical variables were presented as frequencies and numbers (percentages). The Fisher's exact test or chi‐square test was used to compare nominal categorical variables, and the *t*‐test was used for continuous variables. The Kaplan–Meier analysis calculated cumulative survival rates, and survival differences were compared using the log‐rank test. All analysis was performed with SPSS software for Windows (Version 22.0), R (version 4.0.5), and PRISM software (version 8.0, GraphPad Software). A *p*‐value <0.05 were considered statistically significant.

## 3 RESULTS

### 3.1 Demographics and clinicopathologic characteristics

**FIGURE 1:**
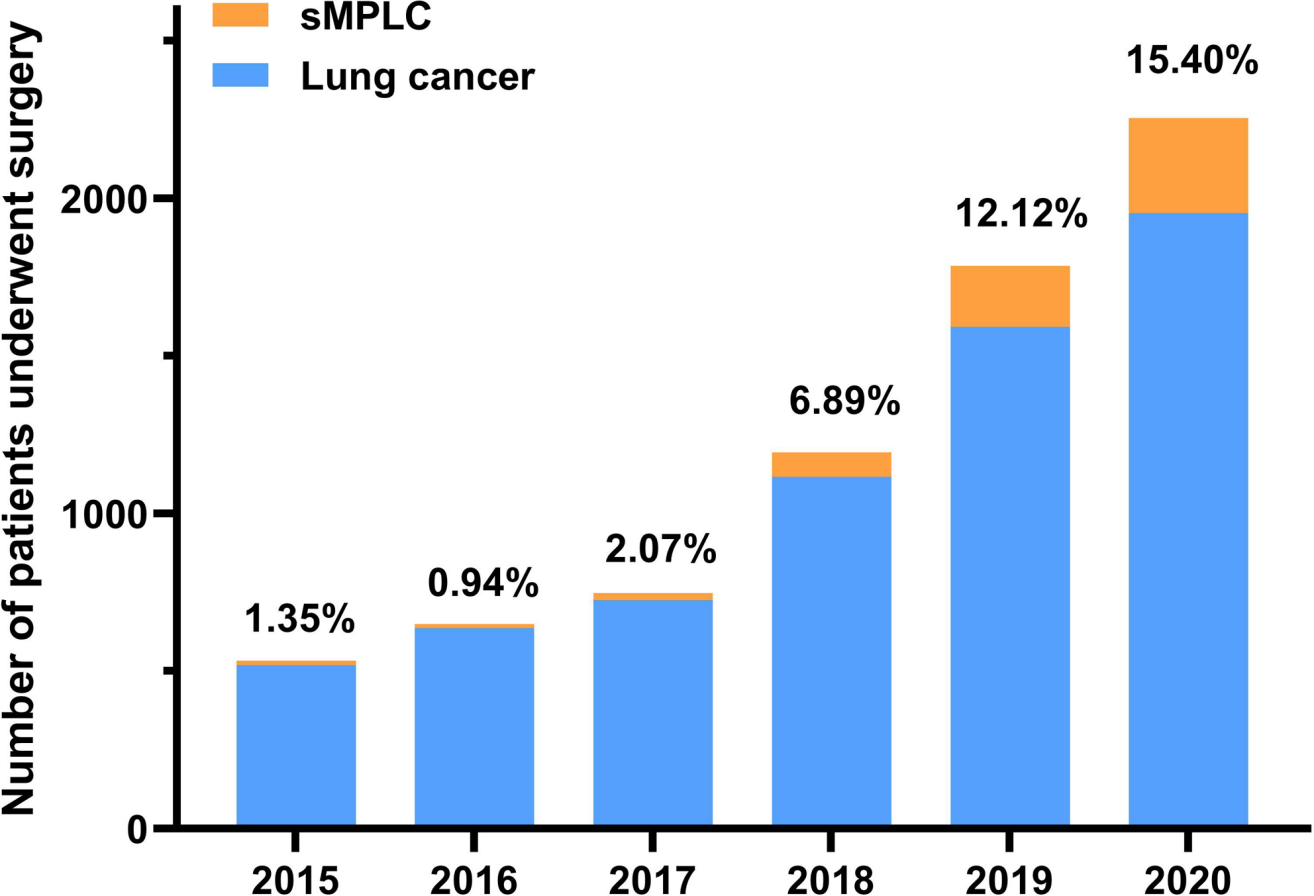
Patients with non‐small cell lung cancer have undergone surgical resection in our Thoracic center since 2015. With the increasing adoption of high‐resolution CT and lung cancer screening programs, the number of patients with lung cancer who underwent surgical treatment increased yearly. Remarkably, patients with early‐stage sMPLC have increased dramatically since 2018.

Among the 582 MPLC patients, 69 patients were diagnosed as metachronous MPLC, and 48 patients underwent lymph node metastasis at the time of surgery. These patients were excluded from the study. In total, there were 465 early‐stage sMPLC patients with 1198 resected lesions included. As shown in Table 1, there were more female (71.2%) and nonsmoker (74.2%) patients, and the median age at initial diagnosis was 55 years old (range 26–78). Most of the lesions were ipsilaterally distributed, presenting as GGO or mGGO. All the patients were pathologically confirmed as sMPLC, and most of the lesions were invasive adenocarcinoma (52.1%, 624/1198) and MIA (26.8%, 321/1198). Only a small number of lesions were diagnosed as other histological types (squamous cell carcinoma (*n* = 9), large‐cell neuroendocrine carcinoma (*n* = 3), adenosquamous carcinoma (*n* = 3), and large‐cell undifferentiated carcinoma (*n* = 2)). Sixty‐seven lesions diagnosed as atypical adenomatous hyperplasia (AAH) were resected as concomitant lesions of the primary lesions. Based on CT findings and clinicopathological results, all 465 patients were diagnosed as T_1‐2_N_0_M_0_ (stage Ia‐Ib) sMPLC patients. Regarding the time of surgery, the number of patients with early‐stage sMPLC has increased dramatically since 2018 (Figure 1).

**TABLE 1:** Clinical characteristics of the sMPLC patients.

Patient characteristics	sMPLC patients (*n* = 1198 lesions/465 pts)
Age (year, IQR)‐no. (%)	55 (26–78)
≤50	132 (28.4%)
>50	333 (71.6%)
Gender (%)	
Male	134 (28.8%)
Female	331(71.2%)
Smoke status‐no. (%)	
Current	56 (12.0%)
Former	54 (11.6%)
Never	345 (74.2%)
N/A	10 (2.2%)
No. of lesions detected per‐patient	
2	305 (65.6%)
3	98 (21.1%)
≥4	62 (13.3%)
Distribution of the lesions‐no. (%)	
In the same lobe	123 (26.4%)
Ipsilateral, different lobe	210 (45.2%)
Bilateral	132 (28.4%)
Tumor location‐no. (%)^a^	
RUL	376 (31.4%)
RML	100 (8.3%)
RLL	244 (20.4%)
LUL	296 (24.7%)
LLL	182 (15.2%)
CT features‐no. (%)^a^	
GGO	669 (55.8%)
mGGO	405 (33.8%)
Solid	124 (10.4%)
Histologic characteristics‐no. (%)^a^	
AAH	67 (5.6%)
AIS	169 (14.1%)
MIA	321 (26.8%)
IAC	624 (52.1%)
Other	17 (1.4%)
Tumor size (cm, IQR)^a^	
0–1.0 cm	838 (69.9%)
1.1–2.0 cm	293 (24.5%)
2.1–3.0 cm	53 (4.4%)
3.0–4.0 cm	14 (1.2%)
Pleural invasion‐no. (%)^a^	
Yes	110 (9.2%)
No	1088 (90.8%)

Abbreviations: AAH, atypical adenomatous hyperplasia; AIS, adenocarcinoma in situ; GGO, ground glass opacity; IAC, invasive adenocarcinoma; IQR, interquartile range; LLL, left lower lobe; LUL, left upper lobe; mGGO, mixed GGO; MIA, minimally invasive adenocarcinoma; N/A, not available; RLL, right lower lobe; RML, right middle lobe; and RUL, right upper lobe.

^a^ Lesion number.

### 3.2 Surgical procedures

Due to the different characteristics of multiple lesions, surgical procedures were performed discriminately. Table 2 demonstrates the surgical procedures in sMPLC. Most patients underwent one‐ or two‐stage VATS or robotic surgery. Only 12 patients underwent thoracotomy due to calcification of lymph nodes, severe adhesion, or abnormal anatomy. Systemic lymph node dissection or sampling was performed according to preoperative pathological results, intraoperative frozen pathology, and lesion characteristics. Lobectomy and segmentectomy were performed to remove the primary lesions, and segmentectomy or wedge resections were applied to remove high‐risk co‐existing lesions according to their size, location, and CT features. Of 333 patients with ipsilateral lesions, multiple lesions underwent removal of all lesions in 181 patients (54.4%) by initial surgery. While of the 132 patients with bilateral lung lesions, 59 patients (44.7%) underwent the removal of all lesions by a one‐stage or two‐stage surgery.

**TABLE 2:** Surgical treatment of sMPLC patients.

Surgery	Ipsilateral, one‐stage surgery (*n* = 333)	Bilateral, one‐stage surgery (*n* = 103)	Bilateral, two‐stage surgery (*n* = 29)	Total
Surgical approach				
VATS	270	92	0	362 (77.8%)
Robotic	51	11	0	62 (13.3%)
Thoracotomy	12	0	0	12 (2.6%)
VATS +VATS	0	0	24	24 (5.2%)
VATS +Robotic	0	0	4	4 (0.9%)
Robotic +Robotic	0	0	1	1 (0.2%)
Surgical procedure				
Lobec	88	2	1	91 (19.6%)
Lobec + Seg	16	4	7	27 (5.8%)
Lobec + Wedge	80	35	6	121 (26.0%)
Lobec +Seg +Wedge	7	4	5	16 (3.4%)
Seg	49	11	4	64 (13.8%)
Seg +Wedge	60	34	4	98 (21.1%)
Wedge +Wedge	33	13	2	48 (10.3%)

Abbreviations: Lobec, lobectomy; Robotic, robotic surgery; Seg, segmentectomy; VATS, video‐assisted thoracoscopic surgery; Wedge, wedge resection.

The average thoracic drainage time was 2.4 ± 3.9 days, the average postoperative hospital duration was 3.2 ± 4.3 days, and the major postoperative complications included pulmonary infection, air leakage, atrial fibrillation, deep vein thrombosis, and incision fat colliquation. All these complications could be significantly improved after symptomatic and supportive treatment. No severe complications or deaths occurred during the perioperative period. All patients were successfully discharged.

### 3.3 Genetic evaluation

**FIGURE 2:**
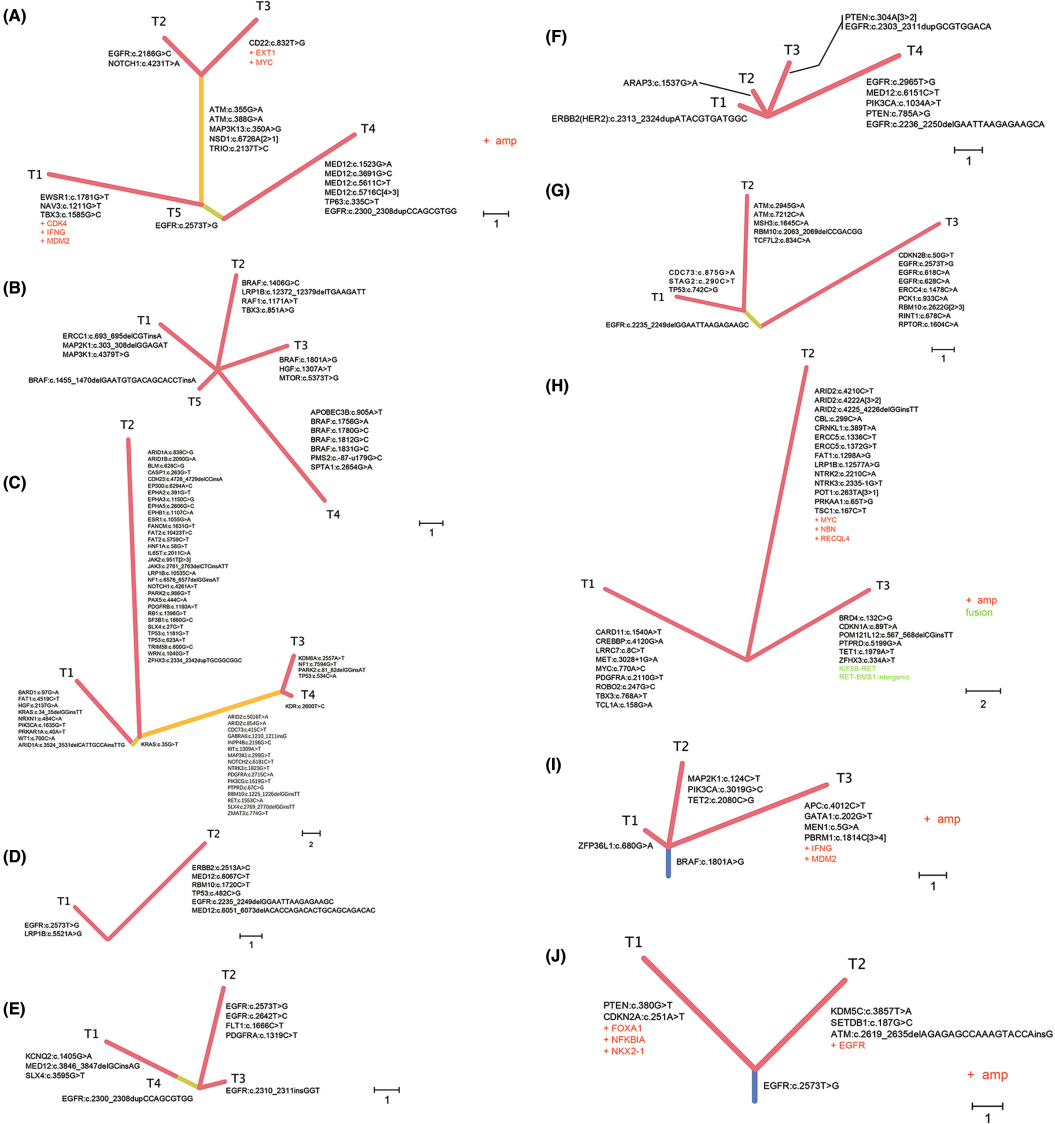
Examples of genetic heterogeneity among the co‐existing lesions in patients with sMPLC. Phylogenetic tree maps demonstrate examples of genetic heterogeneity among co‐existing lesions in patients with sMPLC. Most patients exhibit significant genetic heterogeneity across their multiple tumors. In cases where co‐existing lesions share trunk mutations, histopathological and radiological analyses were re‐evaluated by the multidisciplinary team of thoracic oncology to confirm the sMPLC diagnosis.

A total of 493 lesions from 223 patients underwent wide panel‐genomic sequencing. EGFR was the most common mutated gene and was present in 274 lesions (55.6%). Among them, exon 19 deletions (88/274, 32.1%), L858R missense mutation (101/274, 36.9%), and EGFR amplification (11/274, 4.0%) occurred most frequently. TP53 mutations were found in 76 lesions (15.4%), and high mutation frequency of RBM10 (13.2%), ERBB2 (12.3%), BRAF (9.3%), MED12 (9.1%), and KRAS (7.3%) were also detected. Only 10 lesions (2.0%) harbored EML4‐ALK fusions. As described previously, lesions from sMPLC showed significant genetic heterogeneity.^6^, ^25^ Figure 2 shows examples of genetic heterogeneity among the co‐existing lesions.

Despite most patients with sMPLC being histologically different or harboring different genetic alternations, about 7.5% (35/465) of the patients had the same histological type and driver gene mutation changes. For these patients, radiological and histopathological analyses were re‐evaluated by the multidisciplinary team of thoracic oncology to confirm the sMPLC diagnosis. Lesions presenting as GGO or mGGO or with an element of carcinoma in situ (AIS) on pathological examination were diagnosed as primary lung cancers.^12^, ^26^

### 3.4 Clinical outcomes

**FIGURE 3:**
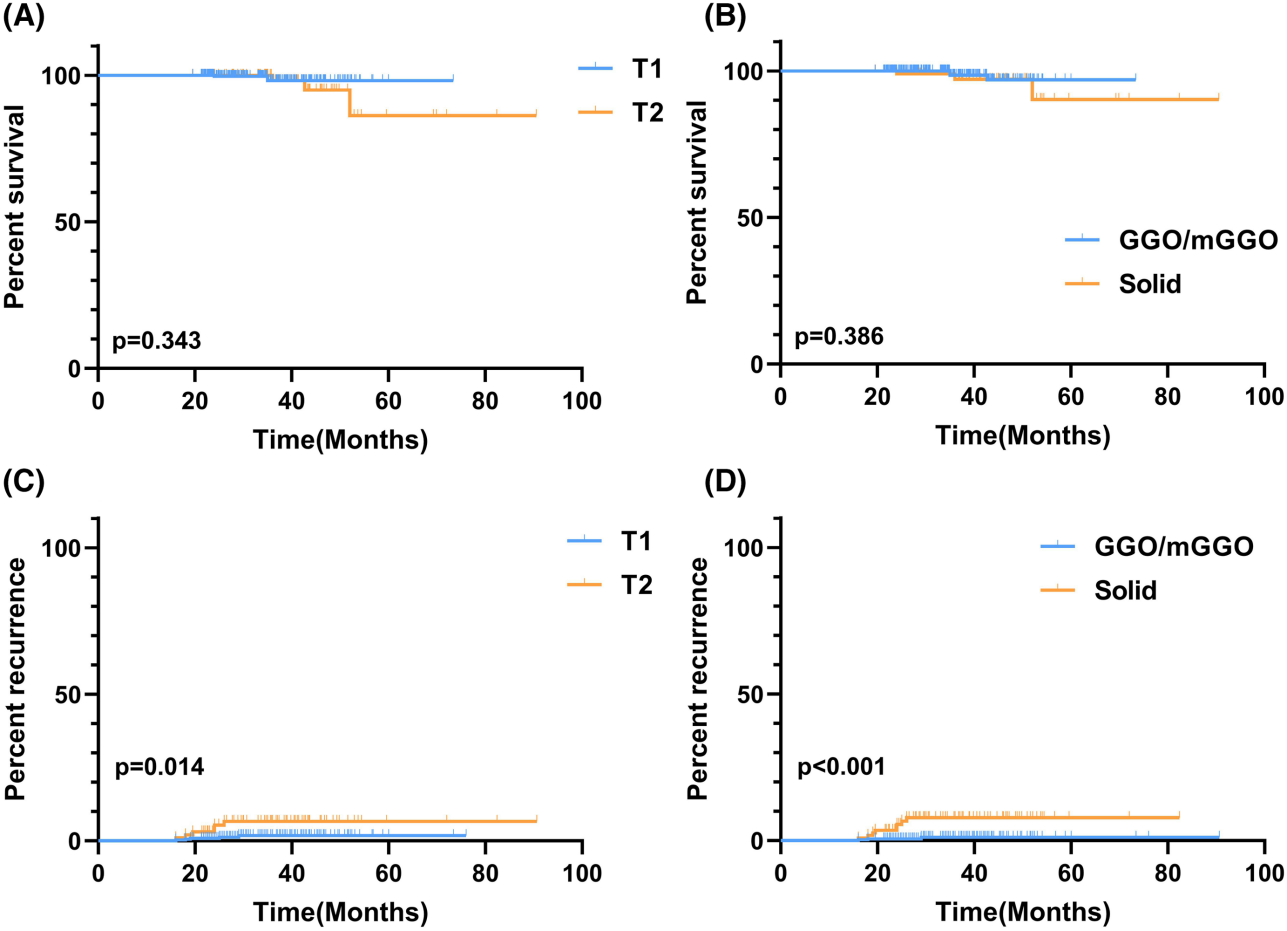
(A, B) Recurrence‐free survival (RFS) of patients with sMPLC. (A) RFS curves between patients with T1 and T2 stage tumors. (B) RFS curves between patients with and without solid lesions. (C, D) Risk factors associated with tumor recurrence. (C) Patients with a T2‐stage primary tumor had a higher tumor recurrence rate. (D) The solid presence of a primary lesion was significantly related to tumor recurrence.

The median follow‐up period was 31.6 months (19.5–90.6 months). There were 11 patients (1.1%) who experienced tumor recurrence or distant metastases, and 6 (1.3%) died at the end of the follow‐up course. There was no significant difference in recurrence‐free survival (RFS) between patients with T1 and T2 stage tumors (*p* = 0.343). The gender, smoking history, CT features, and surgical procedures were unrelated to patients' survival (Figure 3A,B). However, further analyses revealed that the T2 stage (*p* = 0.014, Figure 3C) and the solid presence of a primary lesion (*p* < 0.001, Figure 3D) were significantly related to tumor recurrence.

We next explored the outcomes of the high‐risk residual lesions after the initial surgery. In total, 225 patients had residual lesions after curative‐intent surgery. A progressed residual lesion was defined as the enlargement of the lesion by more than 2 mm or an increased nodule density on the CT scan. The emergence of a new lesion was defined as a newly developed GGO or mGGO nodule after surgery and existed persistently. During the follow‐up period, there were 15 patients with progressed lesions and 28 patients with newly emerged lesions (Table 3). We further investigated the factors associated with residual lesion progression or the emergence of new lesions. The T stage was significantly related to the emergence of new lesions. Patients with a T2‐stage primary tumor had a higher rate of developing new lesions after the initial surgery (*p* < 0.001, Table 4). The age, gender, smoking status, number of lesions, surgical procedures, and CT features were not correlated with the progression of the residual lesions or the emergence of new lesions (Table 4).

**TABLE 3:** Demographics of high‐risk residual or emerged new lesions after the initial surgery.

	Number of lesions/patients (pt. *n* = 225)
Outcome of high‐risk residual lesions^a^	
Stable	203
Progression	15
Dissolution	7
Emergence of new lesions	
No. of lesions/patients	39/28
Size of new lesions (cm)	0.3–1.5
CT feature of new lesions^b^	
GGO	32
mGGO	17
Solid	0
The “X” strategies for high‐risk lesions^c^	
Ablation	43/40
SBRT	8/7
EGFR‐TKI	53/34

^a^ Patient number.

^b^ Lesion number.

^c^ The “X” strategies were applied to treat the high‐risk residual lesions, progressed lesions, and newly emerged lesions with a high possibility of malignancy evaluated by the MDT team.

**TABLE 4:** Univariable and multivariable analysis of risk factors of residual lesion progression or emergence of new lesions.

Variable	Progression of residual lesions				Emergence of new lesions			
	Univariable analysis HR ratio (95% CI)	*p*‐value	Multivariable analysis HR ratio (95% CI)	*p*‐value	Univariable analysis HR ratio (95% CI)	*p*‐value	Multivariable analysis HR ratio (95% CI)	*p*‐value
Age	1.021 (0.982–1.112)	0.653	/	/	2.031 (0.997–3.879)	0.432	/	/
Gender	1.332 (0.845–1.264)	0.745	/	/	1.264 (0.798–1.987)	0.811	/	/
Smoking status	1.597 (1.105–1.894)	0.219	/	/	1.459 (0.687–3.402)	0.398	/	/
No. of lesion	1.024 (0.941–1.059)	0.856	/	/	1.124 (0.780–1.663)	0.989	/	/
Surgical procedure	1.672 (1.083–3.458)	0.710	/	/	0.799 (0.294–1.684)	0.218	/	/
CT features	2.456 (0.779–4.016)	0.064	1.648 (0.892–3.597)	0.229	2.295 (0.976–5.395)	0.065	1.021 (0.845–2.014)	0.156
T stage	2.084 (1.015–3.559)	0.052	2.056 (1.201–3.045)	0.101	6.288 (2.474–15.980)	<0.001	3.048 (1.548–5.884)	<0.001

### 3.5 “X” strategies for high‐risk residual lesions

The “X” strategies, including ablation, SBRT, and EGFR‐TKIs treatment, were applied to treat the high‐risk residual lesions, progressed lesions, and newly emerged lesions with a high possibility of malignancy (Table 3 and Figure S1).

#### 3.5.1 Ablation

CT‐guided percutaneous puncture microwave ablation was applied to 37 patients (with 40 lesions), and 3 patients with three lesions underwent electromagnetic navigation bronchoscopy‐guided microwave ablation (ENB‐MWA). Among the 43 lesions, 32 were GGO lesions, and 11 were mGGO lesions. The median size of the lesions was 1.23 cm (range from 0.6 cm to 2.0 cm). No serious adverse events occurred during the ablation. Two patients suffered mild pneumothorax without symptoms. Lesion responses were evaluated 3 months after ablation. According to RECIST 1.1 criteria,^27^ all the lesions showed complete responses, and no progression was found during the follow‐up course.

#### 3.5.2 SBRT

Seven patients underwent a standard SBRT procedure to treat the high‐risk residual lesions. Six of them had one lesion treated, and one patient had two lesions treated. The SBRT was safe and effective. No severe complications occurred during the treatment.

#### 3.5.3 Postoperative EGFR‐TKI treatment

Thirty‐four patients with 53 residual lesions were treated with postoperative EGFR‐TKI. All these patients were pathologically diagnosed as stage Ib after initial surgery and harbored EGFR mutations in the resected primary lesions. The EGFR‐TKIs treatment was provided according to the latest study results.^20^, ^28^ 13 (24.5%) lesions had responded to the EGFR‐TKI treatment. For the 40 non‐responded lesions, an observation strategy was adopted based on the re‐evaluation of the multidisciplinary team.

## 4 DISCUSSION

The diagnosis and treatment of sMPLC are challenges in clinical practice. Previous studies have demonstrated the safety and efficacy of surgical resection for the primary lesions of sMPLC. However, the alternative strategies for treating high‐risk residual lesions remain controversial. The present study demonstrated real‐world data and outcomes on the clinical diagnosis and comprehensive treatment of early‐stage sMPLC. Several essential findings were present.

First, the safety and efficacy of anatomical resection combined with sublobar resection under a minimally invasive approach were confirmed. Previous studies reported that lobectomy is the optimal choice for the second primary lung cancer, and sublobar resection will not compromise the survival of tumors smaller than 2 cm. However, aggressive surgical treatment, especially pneumonectomy, will bring more comorbidities and worsen the prognosis.^8^, ^9^ The essential surgical treatment of early‐stage sMPLC is to determine the balance between surgical resection and preservation of cardiopulmonary function. On the premise of adequate cardiopulmonary function, anatomical resection combined with sublobar resection was provided to remove the primary lesion and the high‐risk co‐existing lesions as much as possible. No severe complications occurred during the postoperative period. Moreover, the follow‐up data showed a favorite prognosis, and the surgical procedure was not correlated with tumor recurrence or patients' survival, indicating the surgical strategy is safe and effective.

Second, for early‐stage sMPLC, most residual lesions were small GGO or mGGO lesions. According to the evaluation of the multidisciplinary team, a close follow‐up strategy was adopted for most of these residual lesions. The “X” strategies were only provided when a progressed residual lesion or a persistent lesion with a high possibility of invasive adenocarcinoma was found. Recent case‐based studies have reported favorite outcomes of the clinical treatment of residual lesions using ablation and SBRT.^29^, ^30^ Liang and colleagues reported that postoperative EGFR‐TKI treatment would benefit patients with TNM stage III sMPLC that had more than two remaining lesions, mixed component lesions, or diameter of residual lesions ≥8 mm.^31^ In our series of early‐stage sMPLC cases, CT‐guided percutaneous puncture microwave ablation, ENB‐MWA, or SBRT, was provided according to the lesion location, anatomical variations, CT features, and patients' willingness. A high response rate was achieved by ablation and SBRT, and 24.5% of the lesions responded to EGFR‐TKI treatment. Similar to the previous report,^31^ most of the EGFR‐TKI treatment responded lesions were mGGO lesions or diameter of lesions ≥8 mm. No severe toxicity or adverse event was found. Our data demonstrated that, for the treatment of patients with high‐risk residual lesions that were improper for surgical resection, the “X” strategies have their superiority and safety, and the short‐term prognosis was satisfactory.

Third, the previous study has demonstrated that lymph node metastasis was an independent unfavorable prognostic factor of sMPLC.^8^, ^9^ In the present cohort with stage T_1‐2_N_0_M_0_ sMPLC, patients with T2 stage primary tumor or solid primary lesions had a higher rate of tumor recurrence, and a T2 stage primary tumor also significantly related to developing new lesions after the initial surgery, indicating closer follow‐up policy or a more aggressive treatment strategy should be adopted for these patients.

Fourth, it is noteworthy that despite the majority of patients being histologically different or harboring different genetic alterations, about 7.5% of patients had the same histological type and driver gene mutation changes. In this situation, it is rather challenging to differentiate a second primary cancer from a metastatic lesion. The multidisciplinary team re‐evaluated CT features and histopathological analyses to confirm the diagnosis according to previous reports^12^, ^26^: (a) lesions presenting as GGO or mGGO, (b) with an element of AIS on pathologic examination were diagnosed as primary lung cancers. Based on this policy, all these 35 patients were diagnosed as sMPLC, and no tumor recurrence or metastasis was found during the follow‐up course. This finding underscores the importance of a comprehensive evaluation that integrates radiology and pathology, as the sMPLC might have additional genomic and biological characteristics that require further exploration.

There are some limitations of this study. First, this study used a single‐institution Chinese cohort and included surgically resected cases since 2015, an external validation study is needed. Second, for the residual lesions, it is impossible to perform biopsies on every lesion in real‐world practice. We selected the lesions presenting as GGO or mGGO appearance suspected to be malignancy based on the evaluations of the multidisciplinary team of thoracic oncology. Third, the follow‐up period is relatively short for early‐stage sMPLC, especially for the residual lesions after treatment. Long‐term follow‐up will provide more information on the efficacy of the comprehensive treatment. In addition, a small number of patients were lost to follow‐up during the study, which might bias the prognosis results.

In conclusion, our study demonstrated the comprehensive diagnosis and treatment of patients with early‐stage sMPLC in real‐world practice. Histopathological and radiological evaluation combined with genetic analyses could be the robust diagnostic approach for differentiating primary cancer from a metastatic lesion. The “Surgery + X” treatment strategy showed remarkable efficacy, superiority, and safety in the clinical treatment of early‐stage sMPLC.

## AUTHOR CONTRIBUTIONS

**Danting Zhou:** Data curation (equal); investigation (equal); writing – original draft (equal). **Tianyu Yao:** Data curation (equal); writing – original draft (equal). **Xiaojie Huang:** Data curation (supporting); formal analysis (equal); investigation (lead). **Fang Wu:** Project administration (equal); supervision (equal). **Yi Jiang:** Methodology (equal); validation (equal); visualization (equal). **Muyun Peng:** Investigation (equal); project administration (equal); resources (equal); software (equal). **Banglun Qian:** Formal analysis (equal); resources (equal); software (equal). **Wenliang Liu:** Methodology (equal); software (equal); validation (equal). **Fenglei Yu:** Data curation (equal); funding acquisition (equal); resources (equal); supervision (equal); validation (equal); writing – review and editing (supporting). **Chen Chen:** Conceptualization (lead); data curation (equal); funding acquisition (equal); investigation (equal); methodology (lead); Supervision (equal); Writing – review and editing (lead).

## FUNDING INFORMATION

This study was funded by the Hunan Provincial Natural Science Foundation (No. 2020SK53419, 2021JJ30926, and No. 2019JJ50953), Hunan Provincial Key Area R&D Program NO. 2019SK2253, Hunan Clinical Medical Technology Innovation Guidance Project of China Hunan Provincial Science & Technology Department, No. 2020SK53427, CSCO Cancer Research Foundation (CSCO‐Y‐young2019‐034 and CSCO‐2019Roche‐073), and the Changsha Municipal Natural Science Foundation NO. kq2014246.

## CONFLICT OF INTEREST STATEMENT

The authors declare that there are no financial/commercial conflicts of interest involved in this study.

## ETHICS APPROVAL AND CONSENT TO PARTICIPATE

The research protocol of this study was approved by the Institutional Review Board of the Second Xiangya Hospital. Written informed consent was obtained from all subjects.

## CLINICAL TRIAL REGISTRATION NUMBER

Not Applicable.

## Supporting information


Figure S1:
Figure S2:
Figure S3:
Figure S4:

## Data Availability

The datasets generated during the current study are available from the corresponding author upon reasonable request.
