# Supplementary material for: Real‐world comprehensive diagnosis and “Surgery + X” treatment strategy of early‐stage synchronous multiple primary lung cancer
Source: Cancer Med. 2023 Apr 20;12(12):12996–3006. doi: 10.1002/cam4.5972 (PMC10315708; doi:10.1002/cam4.5972)
Supplement: Supplementary file 1 — Figure S1: Figure S2: Figure S3: Figure S4: [file CAM4-12-12996-s001.pdf]

# Supplemental Figure S1-S4. Examples of patients who underwent the "Surgery + X" strategies

**Figure S1.**

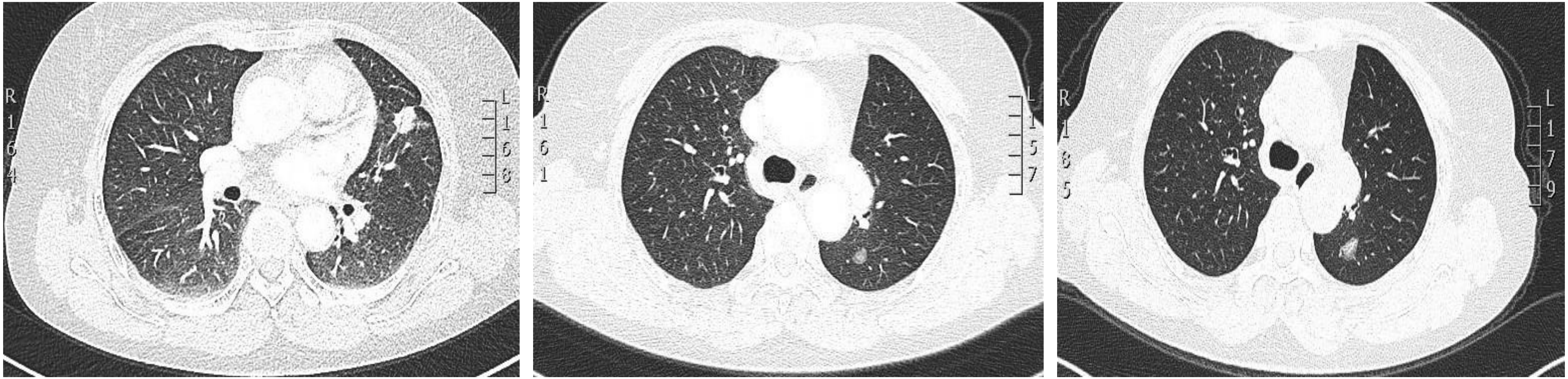

**Primary lesion (2018)** **New emerged lesion (2021)** **Progression (2022)**

**Patient ID: 0033**

57-year-old female.

A lobectomy was performed to remove the primary lesion on the left upper lobe on Aug. 2018. Postoperative pathological diagnosis: adenocarcinoma, $T_2N_0M_0$. A close follow-up strategy was given to observe the residual lesions.

During the follow-up period, a new GGO lesion emerged in left lower lobe (April 2021) and eventually progressed (Feb. 2022).

**Figure S2.**

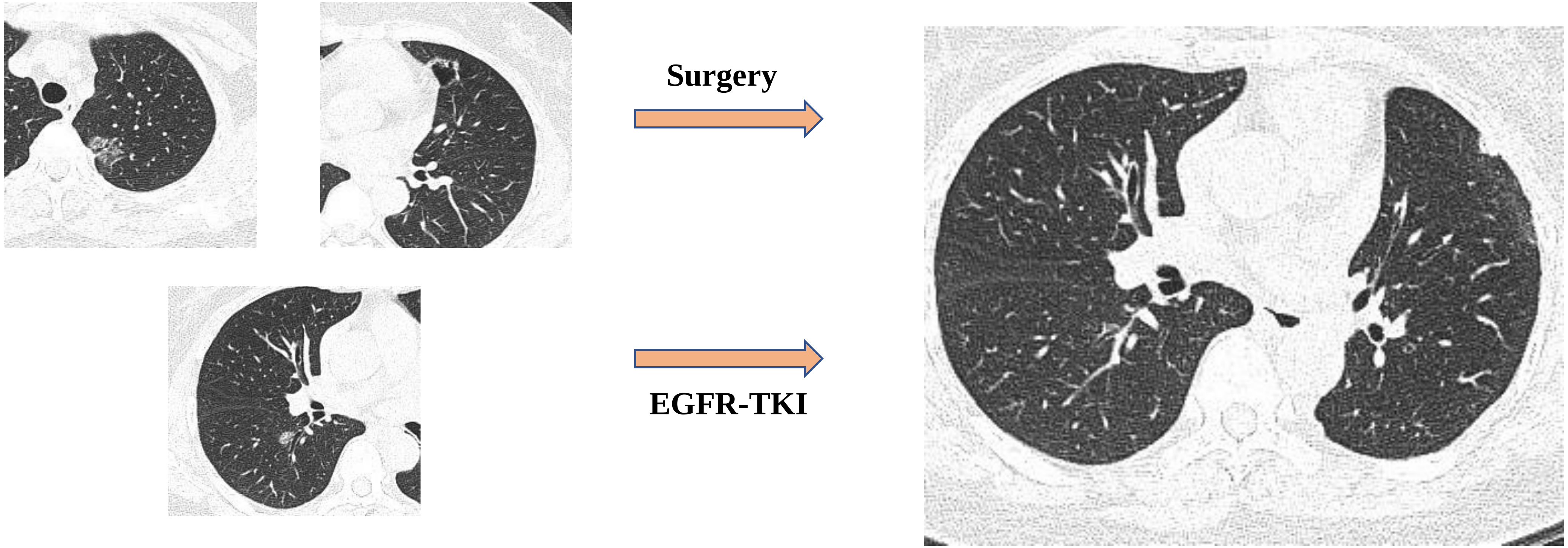


**Multiple lesions (2020)**

**Responded high-risk residual lesion (2022)**

**Patient ID: 0351**

52-year-old female.

A lobectomy was performed to remove the primary lesions on the left upper lobe on Sept. 2020. Postoperative pathological diagnosis: adenocarcinoma, $T_2N_0M_0$, EGFR mutation (L858R). A high-risk residual lesion in the right lower lobe was also identified.

The patient was treated with postoperative EGFR-TKI (Osimertinib). CT scan revealed the high-risk residual lesion has responded to the EGFR-TKI treatment (Agu. 2022).

**Figure S3.**

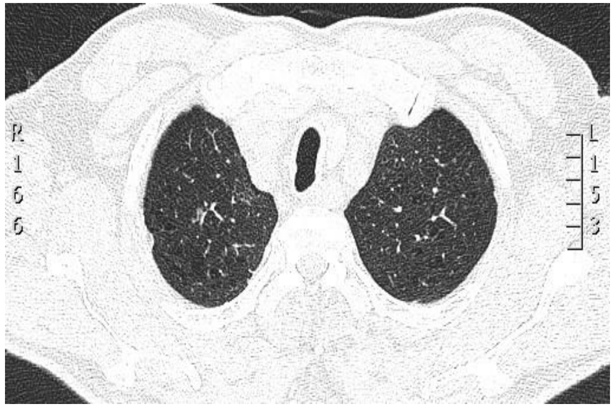

**Residual lesion (2017)**

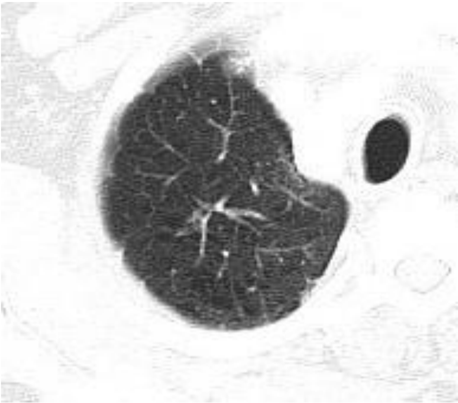

**2019**

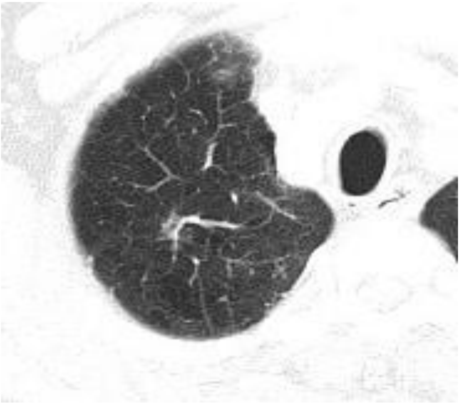

**2020**

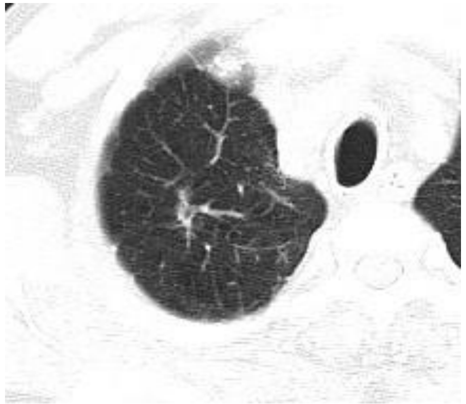

**2021**

**SBRT**

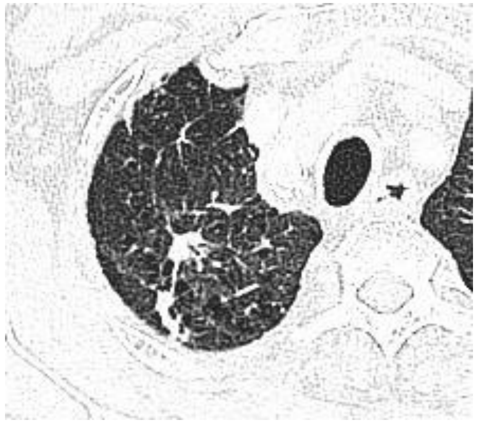

**2022**

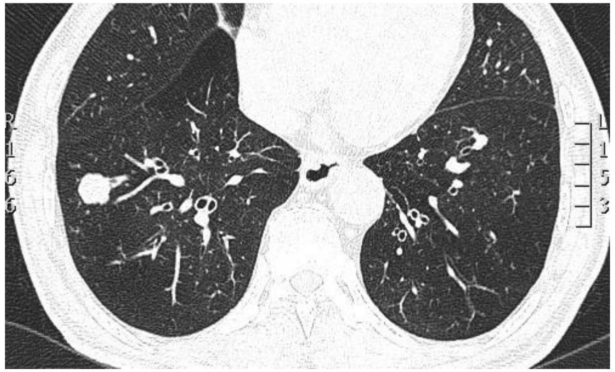

**Primary lesion (2017)**

**Patient ID: 0066**

67-year-old male.

A lobectomy was performed to remove the primary lesion in the right upper lobe on Agu. 2017. Postoperative pathological diagnosis: adenocarcinoma, $T_1N_0M_0$. A follow-up strategy was given to observe the residual lesions.

During the follow-up period, the residual lesion in the right upper lobe eventually progressed and was considered as a high-risk lesion. This lesion was treated by SBRT after evaluation by the MDT team.

**Figure S4.**

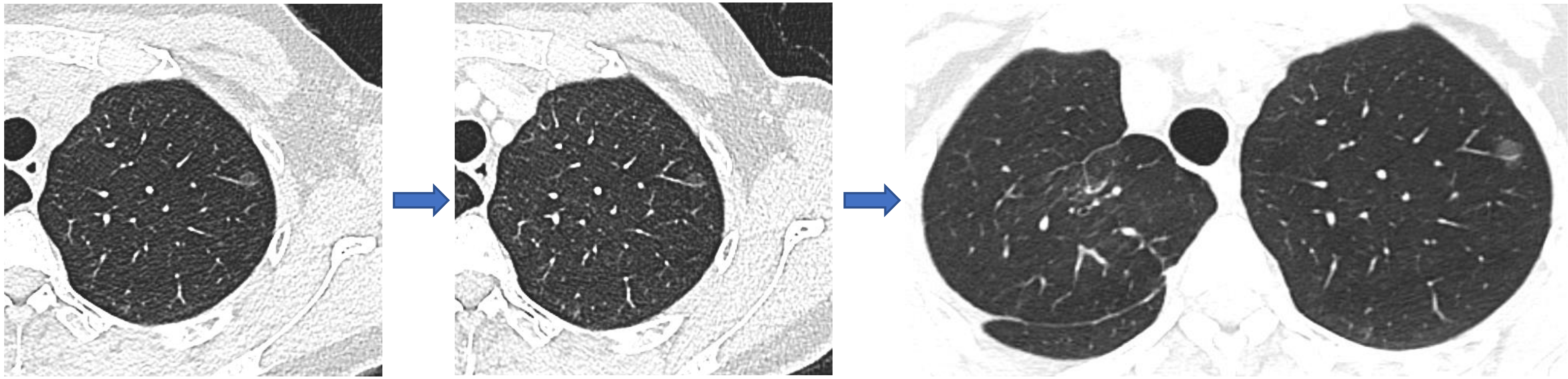

Residual lesion (2019) Residual lesion (2020) Progression (2021)

**Patient ID:0187**

48-year-old female.

A segmentectomy was performed to remove the primary lesion in the right upper lobe on Dec. 2019. Postoperative pathological diagnosis: adenocarcinoma, $T_1N_0M_0$. A follow-up strategy was given to observe the residual lesion in the left upper lobe.

During the follow-up period, the residual lesion in the left upper lobe eventually progressed and was considered as a high-risk lesion. This lesion was treated using CT-guided percutaneous puncture microwave ablation after evaluation by the MDT team.

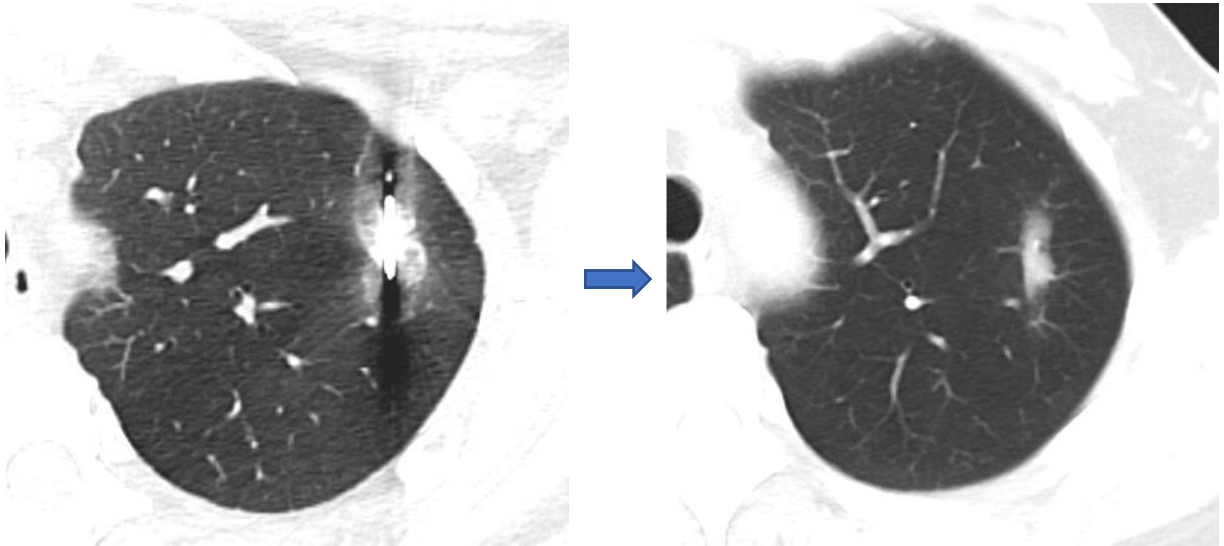

Ablation (2021) Post-ablation (2022)
